# Supplementary material for: The genome sequence of the common crane, Grus grus (Linnaeus, 1758)
Source: Wellcome Open Res. 2025 Mar 4;10:119. [Version 1] doi: 10.12688/wellcomeopenres.23797.1 (PMC11953615; doi:10.12688/wellcomeopenres.23797.1)
Supplement: Peer review history

# Reviewer report: Reviewer response for version 1

**DOI:** 10.21956/wellcomeopenres.26247.r120140

**Published:** 2025-03-28

**Authors:** Bengt Hansson

**Affiliations:** 1 Lund University, Lund, Sweden

This genome assembly report of the common crane ( *Grus grus*) is well-written and contains all necessary information. It presents assembly data of basically every chromosome including the micro-chromosomes and the Z chromosome (the W chromosome was not included in the assembly as a male was sequenced). Before indexing, please check the information of the specimen used for sequencing; in the text it is stated that "The *Grus grus* specimen used for genome sequencing (specimen ID NHMUK014551531, ToLID bGruGru1) was an adult male wild specimen, ...", but Figure 1 shows a chick/juvenile.

Are sufficient details of methods and materials provided to allow replication by others?

Yes

Is the rationale for creating the dataset(s) clearly described?

Yes

Are the datasets clearly presented in a useable and accessible format?

Yes

Are the protocols appropriate and is the work technically sound?

Yes

Reviewer Expertise:

Genomics and population genetics. Birds.

I confirm that I have read this submission and believe that I have an appropriate level of expertise to confirm that it is of an acceptable scientific standard.

# Reviewer report: Reviewer response for version 1

**DOI:** 10.21956/wellcomeopenres.26247.r120137

**Published:** 2025-03-18

**Authors:** Annabel Whibley

**Affiliations:** 1 Bragato Research Institute, Clyde, New Zealand

In this data note, O’Brien, Colom and colleagues present the high-quality genome assembly of the common crane, *Grus grus*.  As a clade where genetic information is already incorporated into conservation management, this more comprehensive reference should provide additional opportunities for implementation of genomic insights in the support of endangered crane species.

 The assembly quality is very good, and the numerous evaluation metrics presented provide a good picture of this. More than 90% of the assembled contigs were assigned to chromosomes. The GC/coverage plot in blobtools shows the characteristic pattern for birds- with macro-chromsomes showing ~40-45% GC  whereas microchromosomes often have a higher GC content. There is more scatter in this plot than is often seen- particularly of small, low-coverage, high GC scaffolds- but these make a very small numerical contribution to the assembly.  The W chromosome is not captured here since the available specimen was male.

 The specimen collection (and identification verified by COI amplification), sequencing and assembly process is carefully and thoroughly documented. RNA sequence data was also obtained but an annotation is not currently provided. The report follows DToL templating, with  publicly available data accessions and full metadata. Recent improvements to this template include more experimental details for HiC, separate reporting of kmer-completeness for the two haplotypes and the inclusion of kmer lengths.

Are sufficient details of methods and materials provided to allow replication by others?

Yes

Is the rationale for creating the dataset(s) clearly described?

Yes

Are the datasets clearly presented in a useable and accessible format?

Yes

Are the protocols appropriate and is the work technically sound?

Yes

Reviewer Expertise:

Genomics Bioinformatics

I confirm that I have read this submission and believe that I have an appropriate level of expertise to confirm that it is of an acceptable scientific standard.
